# Circ-USP9X accelerates deep vein thrombosis after fracture by acting as a miR-148b-3p sponge and upregulates SRC kinase signaling inhibitor 1

**DOI:** 10.1016/j.clinsp.2024.100403

**Published:** 2024-06-14

**Authors:** YongChao Wang, Qin Su, HaiRong Tang, Xin Lin, YanHua Yi, Qiang Tian, ZhangFeng Luo, MeiChun Fu, JiaQi Peng, KeYun Zhang

**Affiliations:** a Department of Joint Sport Medicine, The First Affiliated Hospital of Hunan Medical College, Huaihua City, Hunan Province, PR China; b School of Nursing, Hunan Medical College, Huaihua City, Hunan Province, PR China

**Keywords:** CircUSP9X, miR-148b-3p, Deep vein thrombosis, SRCIN1

## Abstract

**Objectives:**

This study aims to elucidate the role of circUSP9X (Circular RNA Ubiquitin Specific Peptidase 9 X-Linked) in the development of venous thrombosis in the lower extremities.

**Methods:**

An animal model of Deep Vein Thrombosis (DVT) and a hypoxic model of Human Umbilical Vein Endothelial Cells (HUVECs) treated with Cobalt (II) Chloride (CoCl_2_) were developed. The expression levels of circUSP9X, microRNA-148b-3p (miR-148b-3p), and SRC Kinase Signaling Inhibitor 1 (SRCIN1) were quantified using quantitative reverse transcription Polymerase Chain Reaction and Western blot analysis. Cell cytotoxicity, viability, apoptosis, and inflammation in HUVECs were assessed via Lactate Dehydrogenase (LDH) assay, MTT assay, flow cytometry, Enzyme-Linked Immunosorbent Assay, and Western blot, respectively. Hematoxylin and Eosin staining were employed for histopathological examination of the venous tissues in the animal model. The interaction between circUSP9X, miR-148b-3p, and SRCIN1 was further explored through dual-luciferase reporter assays and RNA Immunoprecipitation experiments.

**Results:**

The present findings reveal a significant upregulation of circUSP9X and SRCIN1 and a concurrent downregulation of miR-148b-3p in DVT cases. Knockdown of circUSP9X or overexpression of miR-148b-3p ameliorated CoCl_2_-induced apoptosis in HUVECs, reduced LDH release, enhanced cellular viability, and mitigated inflammation. Conversely, overexpression of circUSP9X intensified CoCl_2_'s cytotoxic effects. The effects of manipulating circUSP9X expression were counteracted by the corresponding modulation of miR-148b-3p and SRCIN1 levels. Additionally, circUSP9X knockdown effectively inhibited the formation of DVT in the mouse model. A competitive binding mechanism of circUSP9X for miR-148b-3p, modulating SRCIN1 expression, was identified.

**Conclusion:**

circUSP9X promotes the formation of DVT through the regulation of the miR-148b-3p/SRCIN1 axis.

## Highlights

• circUSP9X reduction increases cell viability and decreases apoptosis and inflammation in HUVECs
• SRCIN1: A Downstream Target of miR-148b-3p in DVT Pathogenesis.
• SRC Kinase Signaling Inhibitor 1 (SRCIN1) is controlled by miR-148b-3p.

## Introduction

Deep Vein Thrombosis (DVT), as a critical subset of Venous Thromboembolism (VTE), represents a significant global health challenge due to its high mortality rate.^1^ This condition predominantly affects deep veins in the lower leg and thigh, often emerging as a secondary complication post-fracture surgeries.^2^^,^^3^ Despite extensive research, the precise etiological factors and pathogenic mechanisms underlying DVT remain elusive. Studies have consistently highlighted the role of vascular endothelial cell damage and subsequent tissue inflammation under hypoxic conditions as a central feature in DVT development.^4^

In the realm of vascular biology, the emerging role of Circular RNAs (circRNAs), noted for their stability and abundant expression in human tissues, has garnered considerable attention.^5^ These non-coding RNAs, characterized by their unique circular structure, function predominantly within the cytoplasm where they modulate gene expression.^6^ This modulation occurs through the sequestration of microRNAs (miRNAs), thereby influencing the expression of downstream messenger RNAs (mRNAs) and playing a pivotal role in the pathophysiology of various diseases.^7^

Among the diverse array of circRNAs, circUSP9X has emerged as a molecule of interest. Preliminary findings indicate an abnormal upregulation of circUSP9X in patients with DVT compared to healthy individuals, suggesting a potential role in modulating endothelial cell functions and contributing to the pathogenesis of DVT. The intricate interactions of circRNAs, particularly circUSP9X, within the endothelial milieu underscore the complexity of DVT and highlight the need for further investigation. The concept of competing endogenous RNA (ceRNA) networks, wherein circRNAs act as molecular sponges for miRNAs, has been established as a crucial mechanism in disease progression, including DVT.^8^^,^^9^ These networks modulate gene expression by freeing miRNA target mRNAs, thereby increasing their expression levels.^10^ Understanding the role of circUSP9X within these networks could provide valuable insights into the molecular underpinnings of DVT, offering potential avenues for novel therapeutic interventions.

In this study, the authors hypothesize that circUSP9X is intricately involved in the pathogenesis of DVT. The authors aim to elucidate its role and downstream molecular pathways in regulating endothelial cell damage, particularly under hypoxic conditions induced by Cobalt (II) Chloride (CoCl_2_). Furthermore, the authors investigate the impact of circUSP9X modulation on DVT formation in an animal model, paving the way for a deeper understanding of this complex vascular condition.

## Materials and methods

### Patients and specimens

This study received approval from the Ethics Committee of The First Affiliated Hospital of Hunan Medical College (n° 201802HN14). Informed consent was obtained from all participants. Clinical trials were conducted in accordance with the ARRIVE guidelines. Between September 2021 and March 2023, peripheral venous blood samples were collected from 28 patients diagnosed with DVT following knee replacement surgery at The First Affiliated Hospital of Hunan Medical College. Blood samples from all DVT patients were collected within 24 hours post-surgery. According to the Clinical Practice Guidelines of the American College of Chest Physicians,^11^ these fracture patients were diagnosed with lower extremity venous thrombosis using color Doppler ultrasonography and lower limb venography. All DVT patients received a therapeutic dose of low molecular weight heparin (4100 U, twice daily) for chemical prophylaxis post-admission. Post-discharge, prophylactic Rivaroxaban (Bayer, Leverkusen, Germany) at 10 mg daily was administered until 5 weeks post-surgery. Patients were included in the study based on the following criteria: (1) Diagnosed with DVT in lower extremity fractures; (2) No history of DVT in lower extremity fractures; (3) Absence of pathological fractures or cardiac, pulmonary, hepatic, or renal dysfunction. Patients with malignancies, myeloproliferative disorders, common infections, severe autoimmune diseases, or severe psychiatric illnesses were excluded. Additionally, peripheral venous blood samples from 35 healthy subjects were included as controls. Inclusion criteria for healthy subjects were: 1) No history of DVT; 2) Absence of chronic cardiac, pulmonary, hepatic, or renal diseases or other chronic health issues; 3) Age and gender-matched as closely as possible to the DVT patient group; 4) Absence of acute infection or inflammatory symptoms; 5) Generally healthy with no significant medical issues. Exclusion criteria were: 1) Undergoing medication treatments that could affect blood coagulation; 2) Malignancies or diseases affecting the blood; 3) Recent major surgical procedures or trauma. Basic clinical information of the subjects is presented in Table 1. The sample size for the study was calculated using the formula: $n=2\;\times {\left(\frac{Z1-\frac{\alpha }{2}+Z1-\beta }{\delta }\right)}^{2}\times \sigma^{2}$, where α = 0.05, 1-β = 0.8, δ = 0.6, σ = 1. This calculation yielded n = 21.8. Therefore, the sample sizes for both the healthy and DVT groups in this study were deemed sufficient.

**Table 1:** Clinical data.

Characteristic	Healthy subjects	DVT patients
Sample size (n)	35	28
Age (year)		
≥ 60	7	3
< 60	28	25
Gender		
Male	21	17
Female	14	11
BMI		
≤ 18 kg/m^2^	4	7
> 18∼25kg/m^2^	25	16
> 25 kg/m^2^	6	5

### Quantitative reverse transcription PCR (RT-qPCR)

To mitigate RNA degradation, repeated freeze-thaw cycles were strictly avoided in sample handling. RNAse contamination was prevented using SUPERase•In™ RNase Inhibitor (Thermo Fisher Scientific). Total RNA was extracted from human blood samples, murine inferior vena cava tissues, and Human Umbilical Vein Endothelial Cells (HUVECs) using the RNA extraction kit (Invitrogen, CA, USA). Complementary DNA was synthesized from extracted total RNA using the PrimeScript Reverse Transcription Kit. Quantitative PCR for miRNA was performed using the miRNA qPCR Quantitation Kit (GenePharma, Shanghai, China) and SYBR Premix Ex Taq II (Takara, Tokyo, Japan). Glyceraldehyde 3-phosphate dehydrogenase (GAPDH) and U6 were employed as endogenous reference genes. Relative gene expression was calculated using the 2^−ΔΔCt^ method. Primer sequences are listed in Table 2.

**Table 2:** PCR primers.

Genes	Primer sequences (5′–3′)
Human circUSP9X	Forward (F): 5′-GTTGCTCCCAGACTTCATCGC-3′
	Reverse (R): 5′-GACCTTGCTCATCTGGGGGA-3′
Mice circUSP9X	Forward: 5′- TGATCAACAGGTTATGCAATGGT-3′
	Reverse: 5′- ACGAGTCGTGGCTGTCATAC-3′
miR-148b-3p	Forward: 5′- GCGTCAGTGCATCACAGAA-3′
	Reverse: 5′- TGGTGTCGTGGAGTCG-3′
U6	Forward: 5′-CTCGCTTCGGCAGCACA-3′
	Reverse: 5′-AACGCTTCACGAATTTGCGT-3′
Human GAPDH	Forward: 5′-CACCCACTCCTCCACCTTTG-3′
	Reverse: 5′-CCACCACCCTGTTGCTGTAG -3′
Mice GAPDH	Forward: 5′-CATCAACGGGAAGCCCATC-3′
	Reverse: 5′-CTCGTGGTTCACACCCATC-3′

### Cell culture

HUVECs, acquired from the American Type Culture Collection (ATCC; Manassas, VA, USA), were cultured in endothelial cell medium containing 5% Fetal Bovine Serum, 1% penicillin/streptomycin solution (Life Technologies, Paisley, UK), and 10% endothelial cell growth supplement (Sigma-Aldrich, MO, USA). To simulate hypoxia/ischemia, HUVECs were treated with 250 μM CoCl_2_ for 12 hours.

### Actinomycin D and RNase R experiments

For the actinomycin D assay, HUVECs were seeded in six-well plates (4 × 10^5^ cells per well). Twenty-four hours later, cells were exposed to 2 μg/mL actinomycin D (Sigma) and collected at designated time points for RNA stability analysis using RT-qPCR. RNase R (3 U/g, Epicenter) was used to treat RNA (10 μg) from HUVECs, followed by incubation at 37°C for 30 minutes. RT-qPCR was employed to detect circular RNA and linear RNA.

### Cell transfection

siRNAs targeting circUSP9X and SRC1N1, pcDNA 3.1 overexpression vectors, miR-148b-3p mimic, miR-148b-3p inhibitor, and their negative controls were purchased from GenePharma. According to the manufacturer's instructions, these reagents were transiently transfected into HUVECs using Lipofectamine 3000 (Invitrogen). Transfection efficiency was assessed using RT-qPCR and Western blot 48 hours post-transfection. Details of the pcDNA 3.1 overexpression vectors are provided in Supplementary Material 1.

### Lactate Dehydrogenase (LDH) assay

LDH release in the culture supernatant, indicative of cytotoxicity, was assessed using the Pierce LDH Cytotoxicity Assay Kit (Thermo Scientific) as per the kit protocol.

### 3-(4,5-dimethylthiazol-2-yl)-2,5-diphenyltetrazolium bromide (MTT) assay

HUVECs were seeded in a 96-well plate (1 × 10^4^ cells/well) and incubated at 37°C for 24 hours. Cells were then exposed to MTT (10 µL of 5 mg/mL per well) at 37°C for 4 hours. Post-treatment, the solution was removed, and 100 µL of Dimethyl sulfoxide was added to each well to dissolve the formazan product. Finally, the optical density at 570 nm was assessed using a multi-function plate reader (BioTek China) following 15 minutes of shaking as per the manufacturer's protocol.

### Flow cytometry

Apoptosis in HUVECs was detected using the Annexin V/fluorescein isothiocyanate Apoptosis Detection Kit (Southern Biotech, Birmingham, AL, USA). Briefly, cells were harvested, washed with PBS (Invitrogen), and resuspended in a binding buffer. To each sample, 100 μL of cell suspension (1 × 10^6^ cells/mL) was added to 5 μL of Annexin V-FITC (Partec GmbH, CyFlow Space) and 10 μL of Propidium Iodide, incubated at room temperature for 15 minutes, and washed twice with PBS. Cell apoptosis was analyzed using a FACSan flow cytometer (BD Bioscience, Heidelberg, Germany).

### Enzyme-linked immunosorbent assay (ELISA)

Levels of inflammatory cytokines Tumor Necrosis Factor (TNF)-α, Interleukin (IL)-1β, and IL-6 in culture supernatants and tissues were quantified using ELISA Kits as per the manufacturer's instructions (R&D Systems).

### Western blot

Total protein from tissues and cells was extracted using radioimmunoprecipitation assay lysis buffer (Servicebio, Wuhan, China) and quantified with the bicinchoninic acid Protein Assay Kit (Solarbio, Beijing, China). Samples were boiled for 10 minutes with loading buffer, separated on sodium dodecyl sulphate-polyacrylamide gel electrophoresis, and transferred to Polyvinylidene Difluoride membranes. Membranes were blocked (5% non-fat milk in TBST) at room temperature for 2 hours and incubated overnight at 4°C with primary antibodies. This was followed by incubation with immunoglobulin G-Horseradish Peroxidase secondary antibodies for 2 hours, and membranes were washed thrice, 10 minutes each time. Immunoreactivity was detected using the Enhanced Chemiluminescence detection system (Thermo Fisher Scientific, Frederick, MD, USA). Protein bands were analyzed using Image Lab software. Primary antibodies used were as follows: GAPDH (60004-1-Ig, Proteintech), cleaved caspase-3 (ab2302, Abcam), Bax (ab32503, Abcam), Bcl-2 (sc-7382, Santa Cruz Biotechnology), p-p65 (ab86299, Abcam), p65 (ab86299, Abcam), SRCIN1 (ABIN350838, antibodies-online).

### Dual-luciferase reporter assay

Wild-type (WT) 3′-UTR fragments of circUSP9X and SRCIN1 containing predicted miR-148b-3p binding sites were amplified and cloned into the pmirGLO Dual-Luciferase Expression Vector (Promega, Madison, WI, USA) to construct reporter vectors SRCIN1-WT and circUSP9X-WT. The putative binding sites for miR-148b-3p in the 3′-UTR of circUSP9X and SRCIN1 were mutated using the GeneArt™ Site-Directed Mutagenesis PLUS System (cat. no. A14604; Thermo Fisher Scientific, Inc.). Mutant (Mut) 3′-UTRs of circUSP9X and SRCIN1 were cloned into the pmirGLO vector to construct reporter vectors circUSP9X-MUT and SRCIN1-MUT. Reporter vectors and miR-148b-3p mimic or mimic NC were co-transfected into HUVECs using Lipofectamine 3000 reagent. Luciferase activity was measured 48 hours post-transfection using the Dual-Luciferase Reporter Assay System (Promega, Madison, WI, USA).

### RNA Immunoprecipitation (RIP) experiment

RIP was conducted using the RIP Kit (Millipore, Bedford, MA, USA). Briefly, cells were collected and lysed using RIP lysis buffer. Cell extracts were then incubated with RIP buffer containing magnetic beads conjugated with anti-human Ago2 antibody. Proteinase K was applied to digest proteins, and immunoprecipitated RNA was isolated. RNA expression was detected by RT-qPCR.

### DVT animal model

Animal experiments were approved by the Ethics Committee of The First Affiliated Hospital of Hunan Medical College (n° 201808HN61). Clinical trials were conducted in accordance with the ARRIVE guidelines. Efforts were made to minimize animal suffering. Forty C57BL/6J mice (6‒8 weeks old) were obtained from Beijing Vital River Laboratory Animal Technology Co., Ltd. (Beijing, China). Animals were housed in standard laboratory conditions (temperature: 24°±1°C; humidity: 40%‒60%; 12h light-dark cycle) with free access to food and water. After a week of acclimatization, thirty mice were used to establish a DVT model. Briefly, mice were anesthetized with pentobarbital sodium (30 mg/kg, intraperitoneal). The medial thigh hair was removed, and mice were positioned supine. A longitudinal incision was made on the medial thigh to expose the femoral vein 2 cm from the incision. The vein was clamped at three different positions for 30 seconds each with mosquito forceps, followed by suturing the incision. The sham operation group underwent exposure of the inferior vena cava without clamping. Mice were regularly fed post-recovery. Limb swelling and skin color changes were visible on day 1 post-modeling. Venous thrombosis formation in mice was monitored using a high-frequency ultrasound imaging system (Vevo 770, FUJIFILM VisualSonics). To knock down circUSP9X, one week prior to surgery, AVV-shRNA-circUSP9X and control adenovirus AVV-GFP-NC were injected into mice via tail vein (1.6 × 10^11^ vector genomes/mouse). 24 hours post-modeling, mice were euthanized, and inferior vena cava tissues were harvested. Some tissues were fixed in 4% paraformaldehyde, while the rest were frozen at -80°C for further studies.

### Hematoxylin and Eosin (H&E) staining

Collected inferior vena cava tissues fixed in 4% paraformaldehyde were dehydrated using graded alcohols, embedded in paraffin, and sectioned into 4 μm slices. Tissue sections were deparaffinized in xylene and subjected to routine H&E staining. Morphological changes were assessed under a light microscope (LX51, Olympus Optical Co., Ltd, Tokyo, Japan) in five random fields per section.

### Data analysis

Data were analyzed using GraphPad Prism 9.0 software (GraphPad, La Jolla, CA, USA) and presented as mean ± Standard Deviation (SD). Unpaired Student's *t*-test was used to evaluate differences between the two groups. One-way Analysis of Variance (ANOVA) and Tukey's post-hoc test were employed to assess differences among multiple groups. A p-value < 0.05 was considered statistically significant. All experiments in this study were performed with at least three biological replicates.

## Results

### Enhanced circUSP9X expression in DVT context

**Fig. 1:**
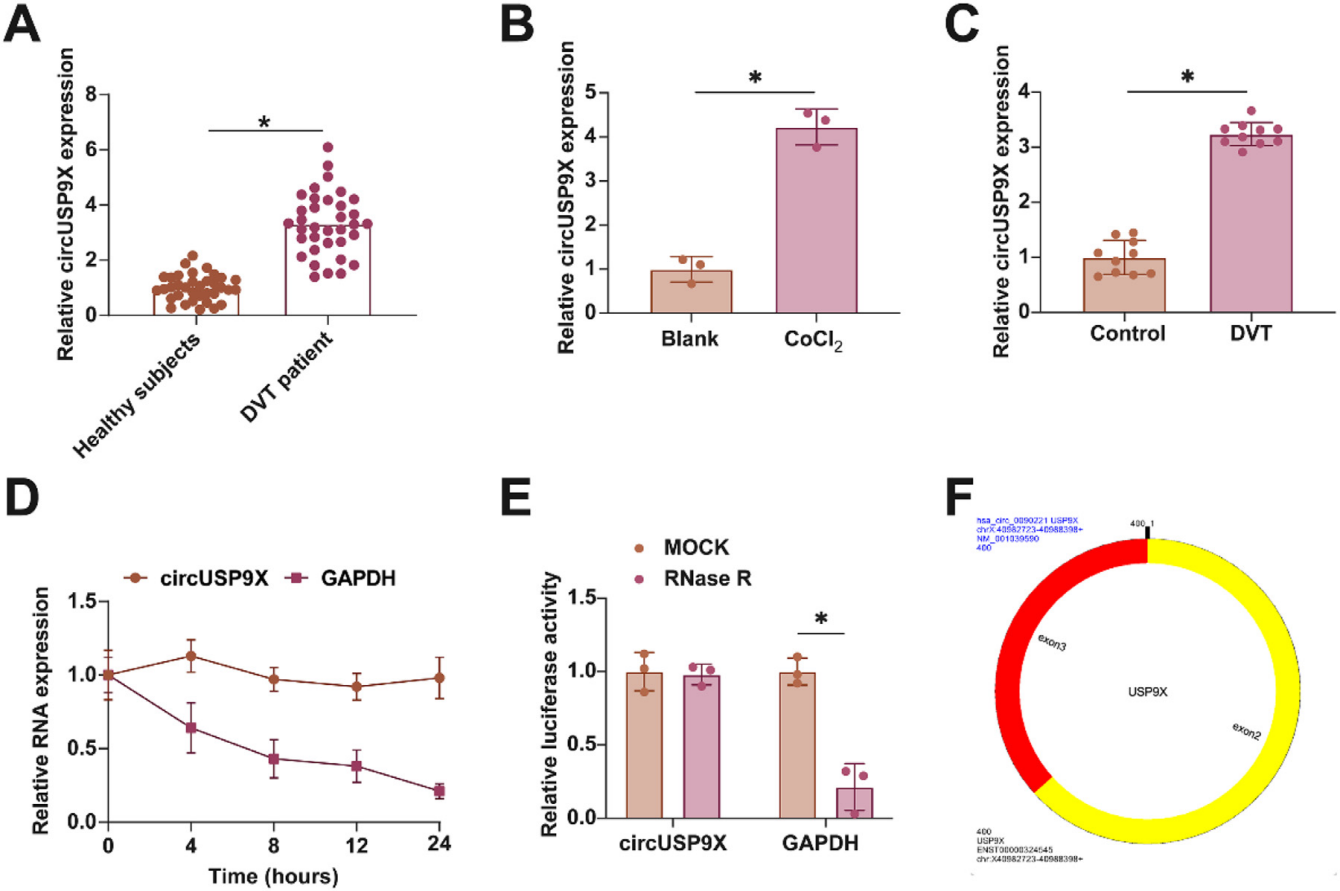
Abnormally high expression of circUSP9X in DVT. (A) RT-qPCR tests of circUSP9X in healthy subjects and DVT patients. (B) RT-qPCR tests of circUSP9X in ClCl_2_-treated HUVECs. (C) RT-qPCR tests of circUSP9X in inferior vena cava tissues of DVT mice. (D) Actinomycin D test of the ring structure of circUSP9X. (E) RNAse R experiment to detect the circUSP9X ring structure; (F) Bioinformatics website circbank for gene information for hsa_circ_0090221; Data are expressed as mean ± SD (C, n = 10; For the rest, n = 3). *p < 0.05.

In examining the role of circUSP9X within the context of DVT, the initial analyses focused on its expression patterns. Fig. 1A reveals that circUSP9X levels are markedly elevated in the blood of DVT patients compared to those in healthy individuals. Additionally, Fig. 1B illustrates that the expression of circUSP9X is significantly augmented in HUVECs following CoCl_2_ treatment. Furthering this exploration, the authors established a DVT mouse model and noted a pronounced increase in circUSP9X expression in the inferior vena cava tissue, as indicated in Fig. 1C. To assess the circular nature of circUSP9X, the authors conducted experiments using Actinomycin D and RNase R. The present findings, depicted in Fig. 1D, show that while Actinomycin D treatment reduces the half-life of GAPDH, it does not affect the stability of circUSP9X. Additionally, RNase R treatment, which digests linear GAPDH mRNA, leaves circUSP9X intact, as shown in Fig. 1E. The authors further referenced the circUSP9X gene information from the biological information website circBank (http://www.circbank.cn), identifying circUSP9X (circBase ID: hsa_circ_0090221) as located on chrX: 40982723-40988398 strand: +, spanning 400 bp and comprising exons 2 and 3 of the USP9X gene (Fig. 1F). These collective data underscore the abnormal elevation of circUSP9X, a circular RNA, in the milieu of DVT.

### Impact of circUSP9X Knockdown on HUVECs: enhanced viability and reduced apoptosis and inflammation

**Fig. 2:**
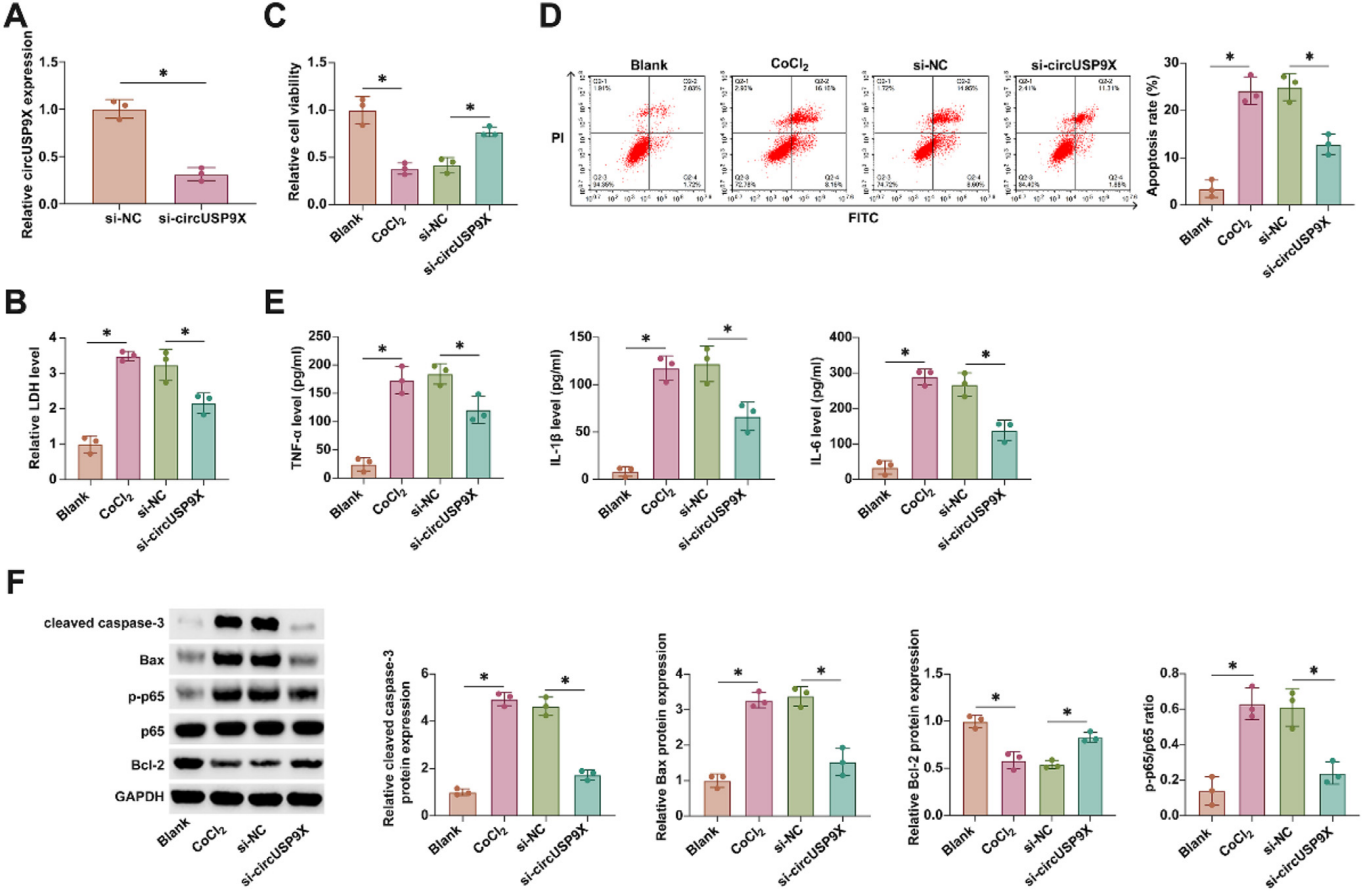
circUSP9X knockdown increases cell viability and decreases apoptosis and inflammation in HUVECs. si-circUSP9X was transfected into CoCl_2_-treated HUVECs. (A) RT-qPCR tests of circUSP9X. (B) Commercial kit to detect LDH release. (C) MTT assay tests of cell viability. (D) Flow cytometry analysis of apoptosis rate. (E) ELISA measurements of IL-1β, IL-6 and TNF-α. (F) Western blot detection of cleaved caspase-3, Bax, p-p65, and Bcl-2; Data expressed as mean ± SD (n = 3). * p < 0.05.

Investigating the functional role of circUSP9X in DVT, the authors employed siRNA targeting circUSP9X (si-circUSP9X) to modulate its expression in HUVECs. As evidenced in Fig. 2A, si-circUSP9X transfection effectively reduced circUSP9X expression. CoCl_2_-induced cytotoxicity, marked by an increase in LDH release, was notably decreased following circUSP9X knockdown, as shown in Fig. 2B. Further, the viability of HUVECs under CoCl_2_ stress, assessed through MTT assays (Fig. 2C), was significantly improved with circUSP9X knockdown. This was accompanied by a reduction in apoptosis rates, depicted in Fig. 2D, indicating a protective effect against CoCl_2_-induced cellular injury. Moreover, ELISA analyses, presented in Fig. 2E, demonstrated a CoCl_2_-driven increase in pro-inflammatory cytokines, which was effectively countered by circUSP9X knockdown. Western blot results, as shown in Fig. 2F, revealed an altered expression of apoptosis-related proteins under CoCl_2_ treatment, which was modulated by circUSP9X knockdown. These findings collectively underscore the potential of circUSP9X as a regulatory factor in endothelial cell dysfunction, suggesting its knockdown as a viable approach to ameliorate cellular injury in the context of DVT.

### Selective Interaction of circUSP9X with miR-148b-3p in DVT

**Fig. 3:**
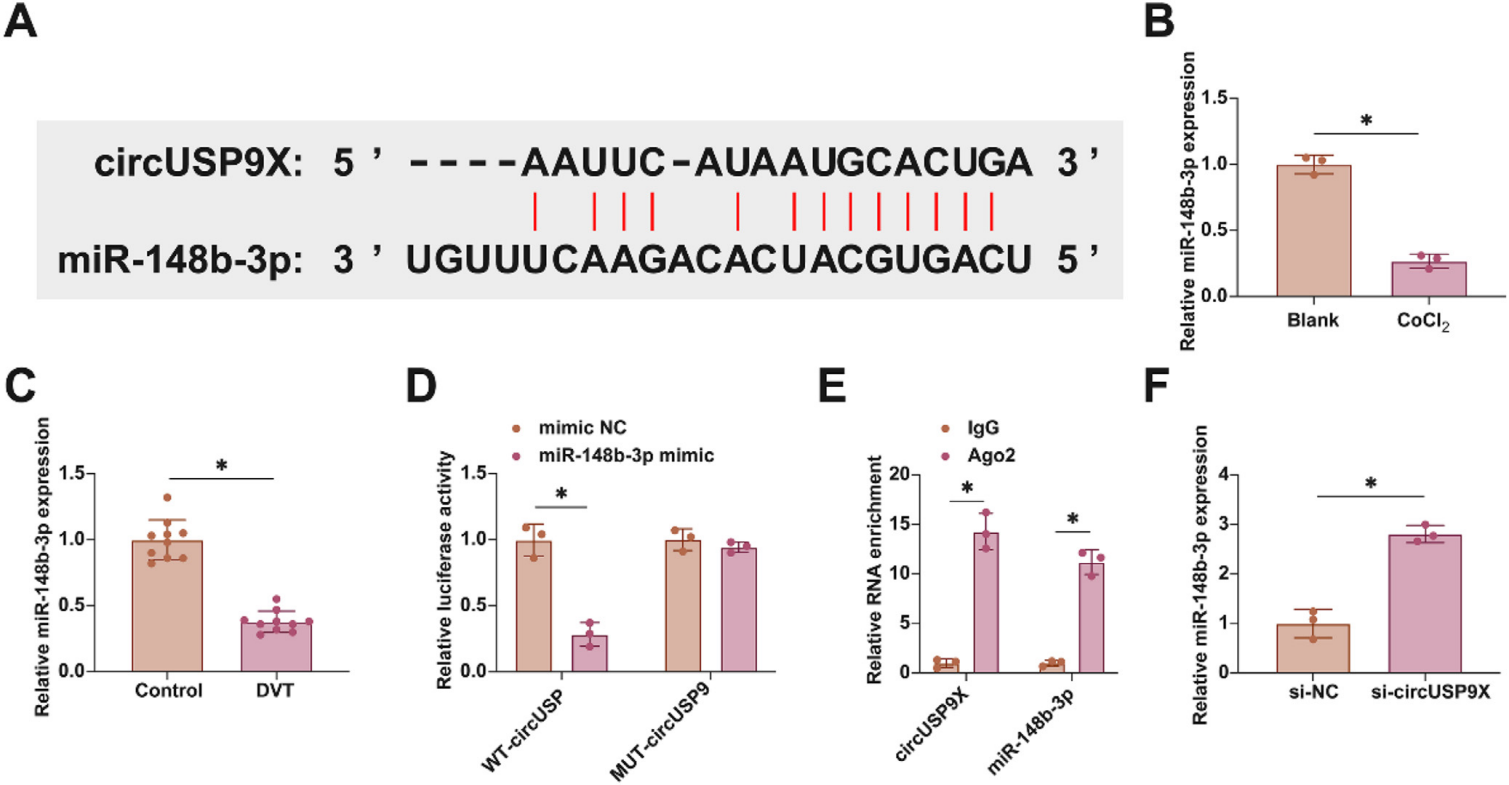
Competitive adsorption of miR-148b-3p by circUSP9X. (A) Starbase predicted the potential binding sites of circUSP9X and miR-148b-3p. (B) RT-qPCR tests of miR-148b-3p in ClCl_2_-treated HUVECs. (C) RT-qPCR tests of miR-148b-3p in inferior vena cava tissues of DVT mice. (D) Dual luciferase reporting assay detection of the targeting binding relationship between circUSP9X and miR-148b-3p. (E) RIP experiment detection of the targeting binding relationship between circUSP9X and miR-148b-3p. (F) RT-qPCR tests of miR-148b-3p; Data are expressed as mean ± SD (C, n = 10; For the rest, n = 3). *p < 0.05.

In exploring the molecular dynamics within DVT, the focus turned to the miRNAs interacting with circUSP9X. Employing the bioinformatics tool starbase for an in-depth analysis, the authors identified miR-148b-3p, known for its protective role against endothelial cell damage, as a key miRNA interacting with circUSP9X. This discovery led us to conjecture a similar function for miR-148b-3p within the DVT framework. Fig. 3A demonstrates potential binding sites between circUSP9X and miR-148b-3p, suggesting a direct molecular interaction. The subsequent studies involved assessing miR-148b-3p's expression patterns in DVT. Fig. 3B and C illustrate a notable decrease in miR-148b-3p expression in HUVECs following CoCl_2_ treatment, a trend also observed in DVT mouse models. To elucidate the binding specificity between circUSP9X and miR-148b-3p, the authors conducted dual-luciferase reporter assays. The results, shown in Fig. 3D, revealed a significant reduction in luciferase activity upon co-transfection with WT-circUSP9X and miR-148b-3p mimic, whereas the MUT-circUSP9X and miR-148b-3p co-transfection did not exhibit such effects. Additionally, RIP experiments highlighted a pronounced enrichment of circUSP9X and miR-148b-3p in Ago2 magnetic beads, as depicted in Fig. 3E. Furthermore, RT-qPCR experiments, presented in Fig. 3F, indicated that the knockdown of circUSP9X led to an upregulation of miR-148b-3p in HUVECs. These findings collectively suggest a targeted regulatory mechanism by circUSP9X on the downstream gene miR-148b-3p.

### circUSP9X modulates cellular toxicity, viability, and apoptosis in HUVECs through miR-148b-3p interaction

**Fig. 4:**
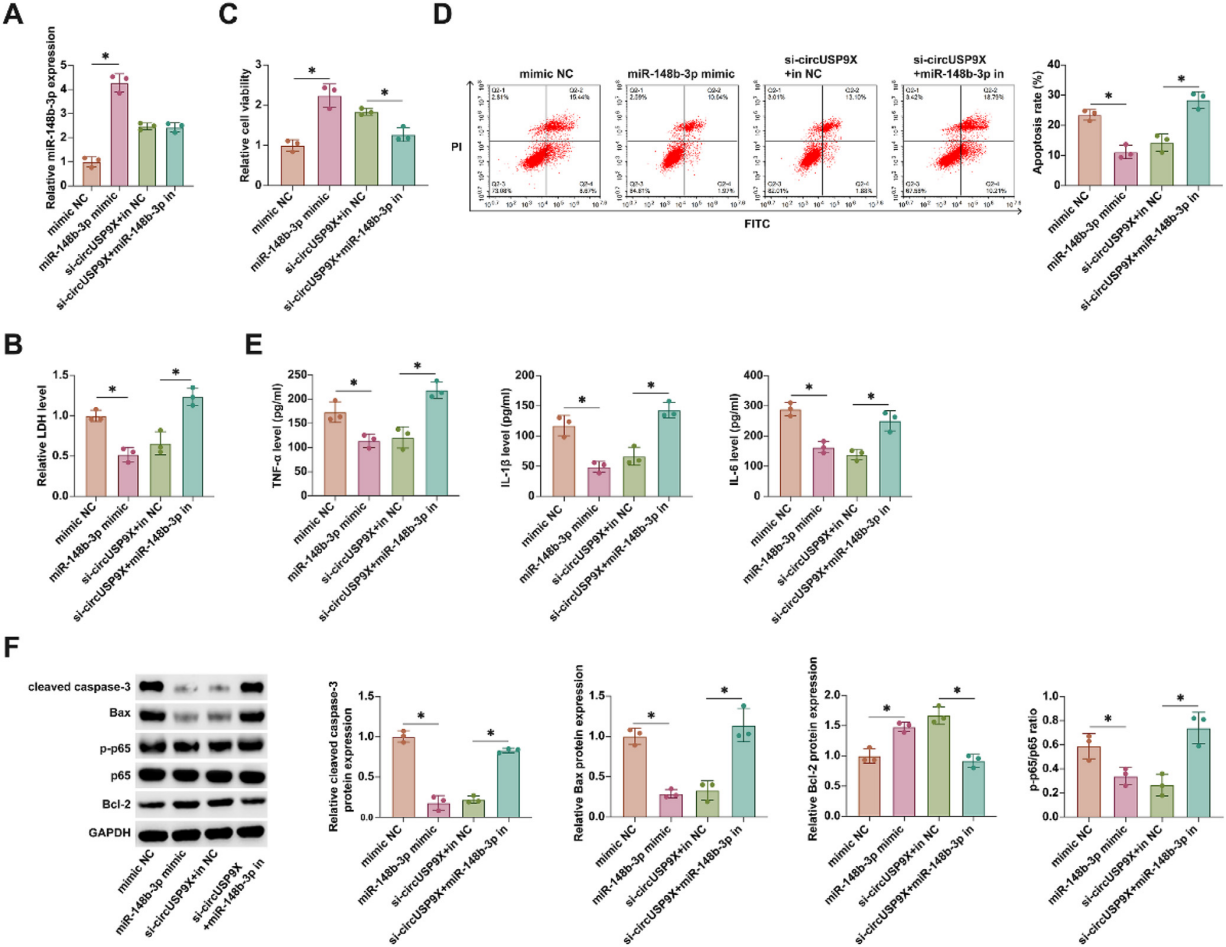
CircUSP9X regulates the cytotoxicity, activity and apoptosis of HUVECs by adsorption of miR-148b-3p. miR-148b-3p mimic was transfected into CoCl_2_-treated HUVECs alone or si-USP9X and miR-148b-3p inhibitor were co-transfected. (A) RT-qPCR tests of miR-148b-3p. (B) Commercial kit to detect LDH release. (C) MTT assay tests of cell viability. (D) Flow cytometry analysis of apoptosis rate. (E) ELISA measurements of IL-1β, IL-6 and TNF-α. (F) Western blot detection of cleaved caspase-3, Bax, p-p65 and Bcl-2; Data expressed as mean ± SD (n = 3). *p < 0.05.

In advancing understanding of miR-148b-3p's role, the authors transfected miR-148b-3p mimic into HUVECs treated with CoCl_2_ and concurrently conducted co-transfection experiments with si-circUSP9X and miR-148b-3p inhibitor in similar conditions. Illustrated in Fig. 4A, transfection with miR-148b-3p mimic and si-circUSP9X substantially increased miR-148b-3p levels. LDH release assays revealed that miR-148b-3p mimics reduced LDH secretion, and notably, the inhibitory effect of si-circUSP9X on LDH release was counteracted by the miR-148b-3p inhibitor, as shown in Fig. 4B. Moreover, Fig. 4C indicates that miR-148b-3p mimics enhanced cellular viability, while the positive impact of si-circUSP9X on cell vitality was attenuated by the miR-148b-3p inhibitor. Flow cytometry analysis, presented in Fig. 4D, demonstrated that miR-148b-3p mimic reduced the rate of cell apoptosis, and this reduction was reversed upon introduction of the miR-148b-3p inhibitor in the presence of si-circUSP9X. ELISA results, as seen in Fig. 4E, indicated that miR-148b-3p mimic decreased the release of inflammatory cytokines, whereas the suppressive effect of si-circUSP9X on these cytokines was negated by the miR-148b-3p inhibitor. Finally, Western blot analysis (Fig. 4F) showed that both miR-148b-3p mimic and si-circUSP9X reduced the expression of cleaved caspase-3, Bax, and p-p65, while increasing Bcl-2 expression. However, these effects of si-circUSP9X were reversed by the miR-148b-3p inhibitor. These findings collectively highlight that circUSP9X regulates key cellular processes in CoCl_2_-treated HUVECs through the modulation of miR-148b-3p.

### SRCIN1: a downstream target of miR-148b-3p in DVT pathogenesis

**Fig. 5:**
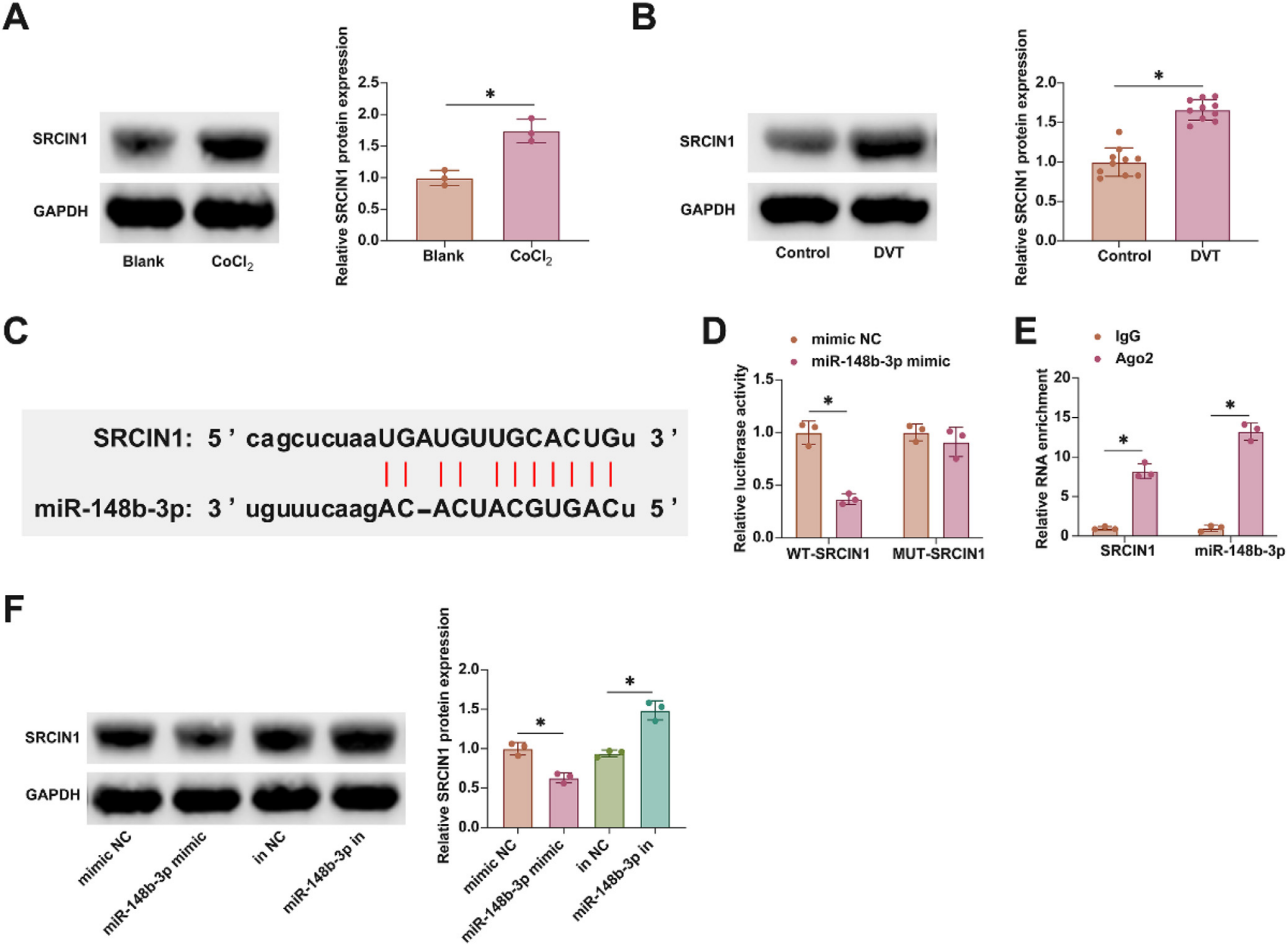
SRCIN1 is the downstream target gene of miR-148b-3p. (A) Western blot tests of SRCIN1 in ClCl_2_-treated HUVECs. (B) Western blot tests of SRCIN1 in inferior vena cava tissues of DVT mice. (C) Starbase predicted the targeted binding sites of miR-148b-3p and SRCIN1. (D) Dual luciferase reporting assay detection of the targeting binding relationship between SRCIN1 and miR-148b-3p. (E) RIP experiment detection of the targeting binding relationship between SRCIN1 and miR-148b-3p. (F) Western blot tests of SRCIN1. Data are expressed as mean ± SD (n = 3). *p < 0.05.

In furthering this investigation into the molecular mechanisms of DVT, attention was focused on the downstream targets of miR-148b-3p. Notably, SRCIN1 has been implicated in DVT, exhibiting aberrant expression patterns, a finding corroborated by the present study as illustrated in Fig. 5A and B. Utilizing the starbase bioinformatics portal, the authors identified putative interaction sites between miR-148b-3p and SRCIN1, as shown in Fig. 5C. Subsequent experimental validation using dual-luciferase reporter assays and RIP experiments affirmed this interaction. Co-transfection with WT-SRCIN1 and miR-148b-3p mimic led to a notable reduction in luciferase activity. Additionally, SRCIN1 and miR-148b-3p were found to be enriched in Ago2 magnetic beads, as presented in Fig. 5D and E. Moreover, the modulation of miR-148b-3p levels, either through overexpression or knockdown, inversely affected SRCIN1 protein expression, as evidenced in Fig. 5F. These findings elucidate SRCIN1 as a key downstream target of miR-148b-3p.

### Exacerbation of CoCl_2_ toxicity in HUVECs by overexpressed circUSP9X and its reversal by SRCIN1 knockdown

**Fig. 6:**
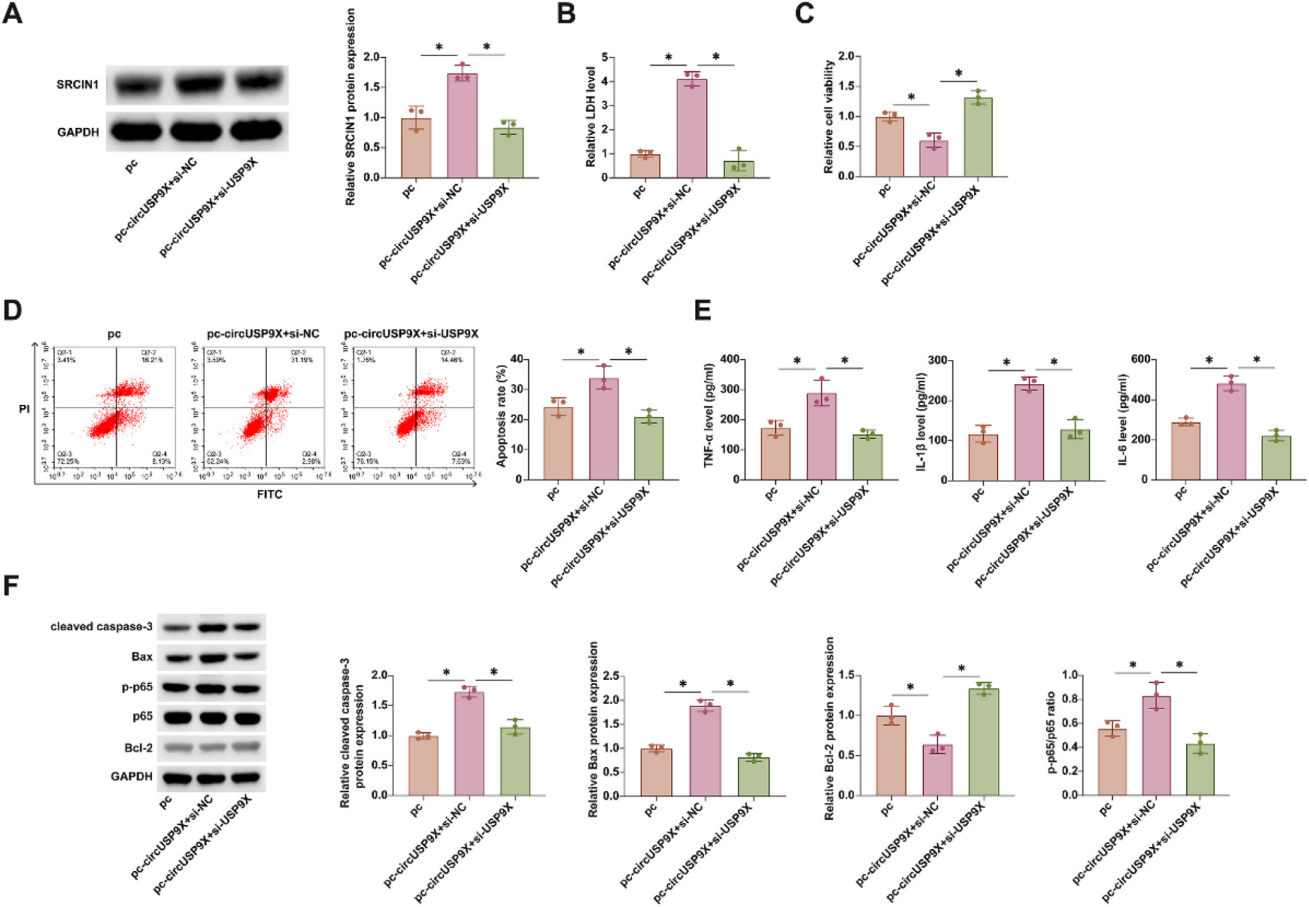
Overexpressing circUSP9X increases CoCl_2_ toxicity to HUVECs, but this effect is inhibited by knocking down SRCIN1. pcDNA 3.1-circUSP9X and si-SRCIN1 were co-transfected into CoCl2-treated HUVECs. (A) Western blot tests of SRCIN1. (B) Commercial kit to detect LDH release. (C) MTT assay tests of cell viability. (D) Flow cytometry analysis of apoptosis rate. (E) ELISA measurements of IL-1β, IL-6 and TNF-α. (F) Western blot detection of cleaved caspase-3, Bax, p-p65 and Bcl-2; Data expressed as mean ± SD (n = 3). * p < 0.05.

In these subsequent experiments, the authors co-transfected pcDNA 3.1-circUSP9X and si-SRCIN1 into HUVECs treated with CoCl_2_. Fig. 6A shows that overexpression of circUSP9X via pcDNA 3.1-circUSP9X enhanced SRCIN1 protein expression, an effect that was reversed upon SRCIN1 knockdown. Rescue experiments further revealed that overexpression of circUSP9X increased LDH release, reduced cellular viability, promoted apoptosis, and elevated the release of inflammatory cytokines TNF-α, IL-1β, and IL-6. Additionally, it upregulated cleaved caspase-3, Bax, and p-p65 protein expression while suppressing Bcl-2 expression. Notably, all these effects were reversed by the knockdown of SRCIN1, as depicted in Fig. 6B‒F. These findings underscore SRCIN1 as a functional protein in the circUSP9X-mediated regulatory pathway in DVT, highlighting its potential role as a key modulator of endothelial cell toxicity.

### Knockdown of circUSP9X mitigates venous thrombosis in a murine model

**Fig. 7:**
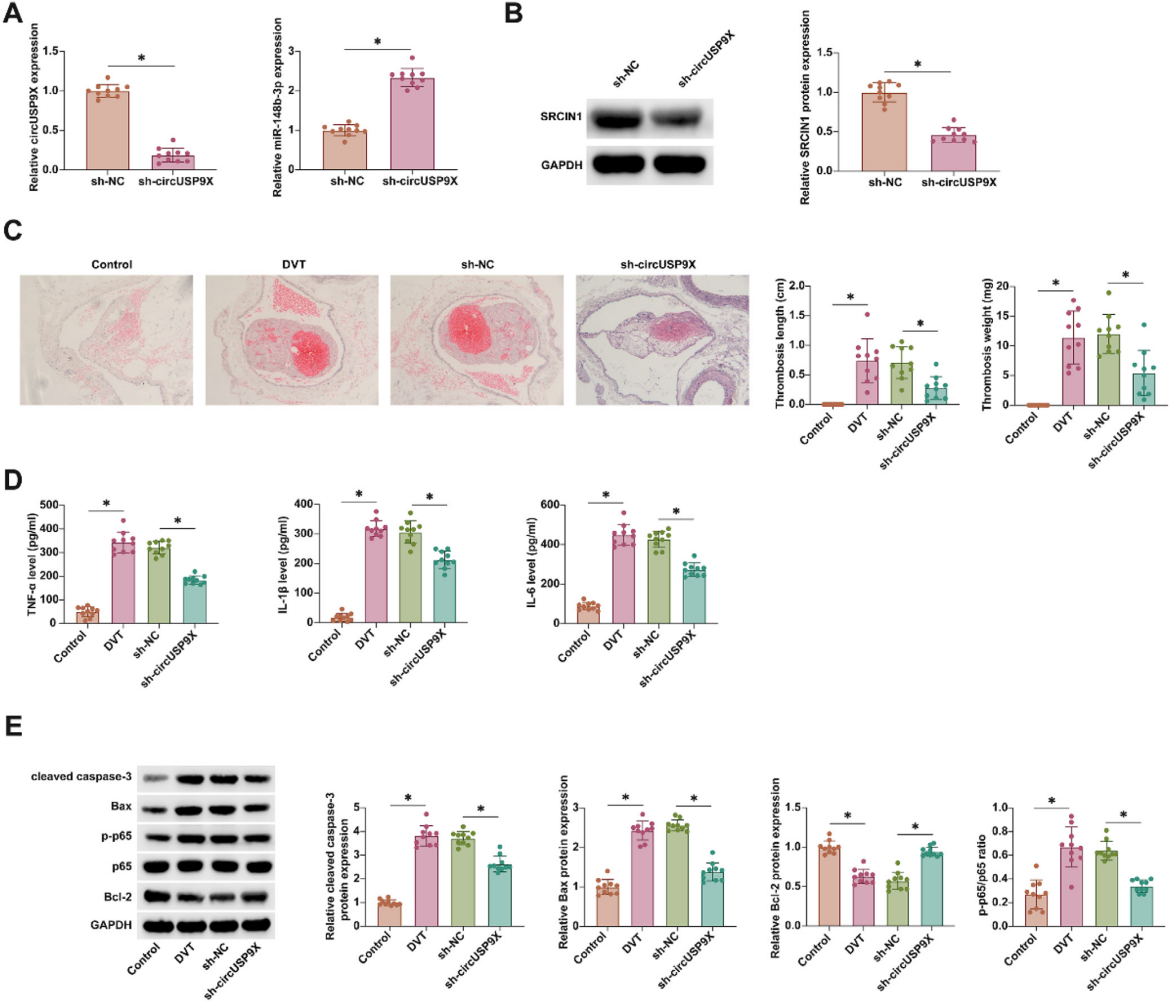
Knocking down circUSP9X improves DVT *in vivo*. (A) RT-qPCR tests of circUSP9X and miR-148b-3p in blood of mice. (B) Western blot tests of SRCIN1 in inferior vena cava tissues of mice. (C) HE-staining representative images of inferior vena cava tissue of mice, thrombus length and thrombus weight; (D) ELISA measurements of IL-1β, IL-6 and TNF-α. (F) Western blot tests of cleaved caspase-3, Bax, p-p65 and Bcl-2; Data are expressed as mean ± SD (n = 10). *p < 0.05.

In the *in vivo* investigation of circUSP9X's role in DVT, a DVT mouse model was employed. Results from RT-qPCR and Western blot analyses illuminated that circUSP9X knockdown effectively diminished SRCIN1 expression while concurrently augmenting miR-148b-3p expression (Fig. 7A and B). HE-staining further illustrated the impact of circUSP9X knockdown, revealing a marked reduction in thrombus formation in DVT mice (Fig. 7C). ELISA assays (Fig. 7D) showed a pronounced decrease in the levels of inflammatory cytokines in the inferior vena cava tissues of mice with circUSP9X knockdown. Additionally, Western blot analysis (Fig. 7E) confirmed that this genetic intervention led to a notable suppression of cleaved caspase-3, Bax, and p-p65 proteins, alongside an increase in Bcl-2 expression. These collective findings underscore the therapeutic potential of circUSP9X knockdown in mitigating venous thrombosis.

## Discussion

DVT involves the interaction of vascular endothelial cells, platelets, and clotting-related proteins.^12^ DVT is asymptomatic and is easily overlooked in its early stages.^13^ Apoptosis and inflammation of vascular endothelial cells can be observed during DVT formation.^14^ Therefore, VEC injury is an important reason for DVT. Here, HUVECs were exposed to CoCl_2_ to simulate hypoxia during DVT, and circUSP9X's role in hypoxia injury in HUVECs was probed. Finally, it was delineated that circUSP9X increased CoCl_2_-mediated cytotoxicity, increased apoptosis and inflammation, and decreased cell viability by competitively adsorbing miR-148b-3p and mediating SRCIN1 expression.

It is a major problem to detect DVT early. circRNA can be used as an early diagnostic biomarker for a variety of diseases, such as gestational diabetes,^15^ cancer,^16^ and osteoarthritis.^17^ This study noted that circUSP9X was forced in the peripheral blood of fracture patients with DVT. The same trend was observed in the blood of mice with DVT modeling. This suggests that abnormally expressed circUSP9X may serve as a potential biomarker for DVT, which will be beneficial for the early detection and treatment of DVT. However, ROC curve analysis is needed to prove the possibility of circUSP9X as a biomarker of DVR in subsequent studies.

Apoptosis and inflammation of VECs are significant causes of DVT. It is discussed that circUSP9X exacerbates oxidized Low-Density Lipoprotein (ox-LDL)-induced HUVECs damage, which includes apoptosis, inflammation, and oxidative stress.^18^^,^^19^ This study further indicated that circUSP9X controlled endothelial cell function. In detail, knocking down circUSP9X effectively reduced CoCl_2_-induced apoptosis and inflammation of HUVECs and increased cell viability. As suggested, Nuclear Factor (NF)-κB pathway hyper-activation accelerates DVT formation.^20^ Therefore, inhibiting NF-κB phosphorylation by knockdown of circUSP9X may be an important reason for reducing tissue inflammatory factors and accelerating thrombolysis. miR-148b-3p/SRCIN1 axis was confirmed as the downstream target of circUSP9X. MiR-148b-3p mediates VEC activity and VEC damage. It can protect against ox-LDL, and oxygen-glucose deprivation/reoxygenation-induced VEC injury.^21^^,^^22^ The present results also confirm the beneficial role of miR-148b-3p in protecting against VEC damage. Overexpressing miR-148b-3p effectively improved CoCl_2_-induced HUVEC inflammation and apoptosis. Mechanistically, miR-148b-3p acted by regulating the downstream gene SRCIN1. SRCIN1 triggered the activation of SRC tyrosine Kinase (Csk), which is a member of a family of non-receptor tyrosine kinase proteins and plays a crucial role in angiogenesis.^23^^,^^24^ It has been documented that the activity of SRC is markedly diminished in HUVECs exposed to ox-LDL, and the pro-angiogenic effects of miR-150 mimic in HUVECs exposed to ox-LDL are entirely nullified in the presence of an SRC inhibitor. Upregulation of miR-150 promoted angiogenesis and proliferation of endothelial progenitor cells by targeting SRCIN1 *in vitro* and thrombus resolution *in vivo*.^25^

In conclusion, this study suggests that circUSP9X is a key regulator of CoCl_2_-induced HUVEC dysfunction. It affects cytotoxicity, apoptosis, and inflammation of HUVECs by adsorbing miR-148b-3p and mediating SRCIN1. Moreover, knocking down circUSP9X can promote the decomposition of DVT. The results of this study provide a new molecular target for DVT-targeting drugs.

## Ethics approval

The present study was approved by the Ethics Committee of The First Affiliated Hospital of Hunan Medical College (n° 201802HN14) and written informed consent was provided by all patients prior to the study start. All procedures were performed in accordance with the ethical standards of the Institutional Review Board and The Declaration of Helsinki, and its later amendments or comparable ethical standards.

And the animal experiment research protocol was approved by The First Affiliated Hospital of Hunan Medical College (n° 201808HN61) and performed in accordance with the “Guidelines for the care and use of experimental animals”.

## Authors’ contributions

Conceptualization, KeYun Zhang and Qin Su; methodology, HaiRong Tang, Qiang Tian and Xin Lin; formal analysis, ZhangFeng Luo and MeiChun Fu; investigation, JiaQi Peng and HongTao Zhao; data curation, YongChao Wang; writing-original draft preparation, KeYun Zhang and Qin Su; writing-review and editing, YongChao Wang; project administration, YongChao Wang. All authors have read and agreed to the published version of the manuscript.

## Funding

1) Hunan Provincial Natural Science Foundation of the general project, Differences and molecular mechanisms of postoperative lower limb VTE formation in elderly patients with hip fractures of different blood types (n° 2020JJ4061).
2) 2022 annual project of Hunan Disabled Persons' Rehabilitation Association, Research on the Effect of Virtual Reality Technology (VR) Combined with Ontological Function Training on the Rehabilitation of Limb Function in Children with Cerebral Palsy (n° 2022XK0223)
3) Hunan Provincial Innovation Platform and Talent Plan, Hunan Province Multiple Severe Trauma Treatment Clinical Medical Technology Demonstration Base (n° 2019SK4019)

## Declaration of competing interest

The authors declare no conflicts of interest.

## Data Availability

The datasets used and/or analyzed during the present study are available from the corresponding author upon reasonable request.

## Appendix Supplementary materials

Image, application 1
