# Supplementary figures and images for: Circ-USP9X accelerates deep vein thrombosis after fracture by acting as a miR-148b-3p sponge and upregulates SRC kinase signaling inhibitor 1
Source: Clinics (Sao Paulo). 2024 Jun 14;79:100403. doi: 10.1016/j.clinsp.2024.100403 (PMC11226750; doi:10.1016/j.clinsp.2024.100403)

**CLINICS-D-23-00725 – Supplementary Material**

**Figure Supplementary** pcDNA 3.1 plasmid vector map.


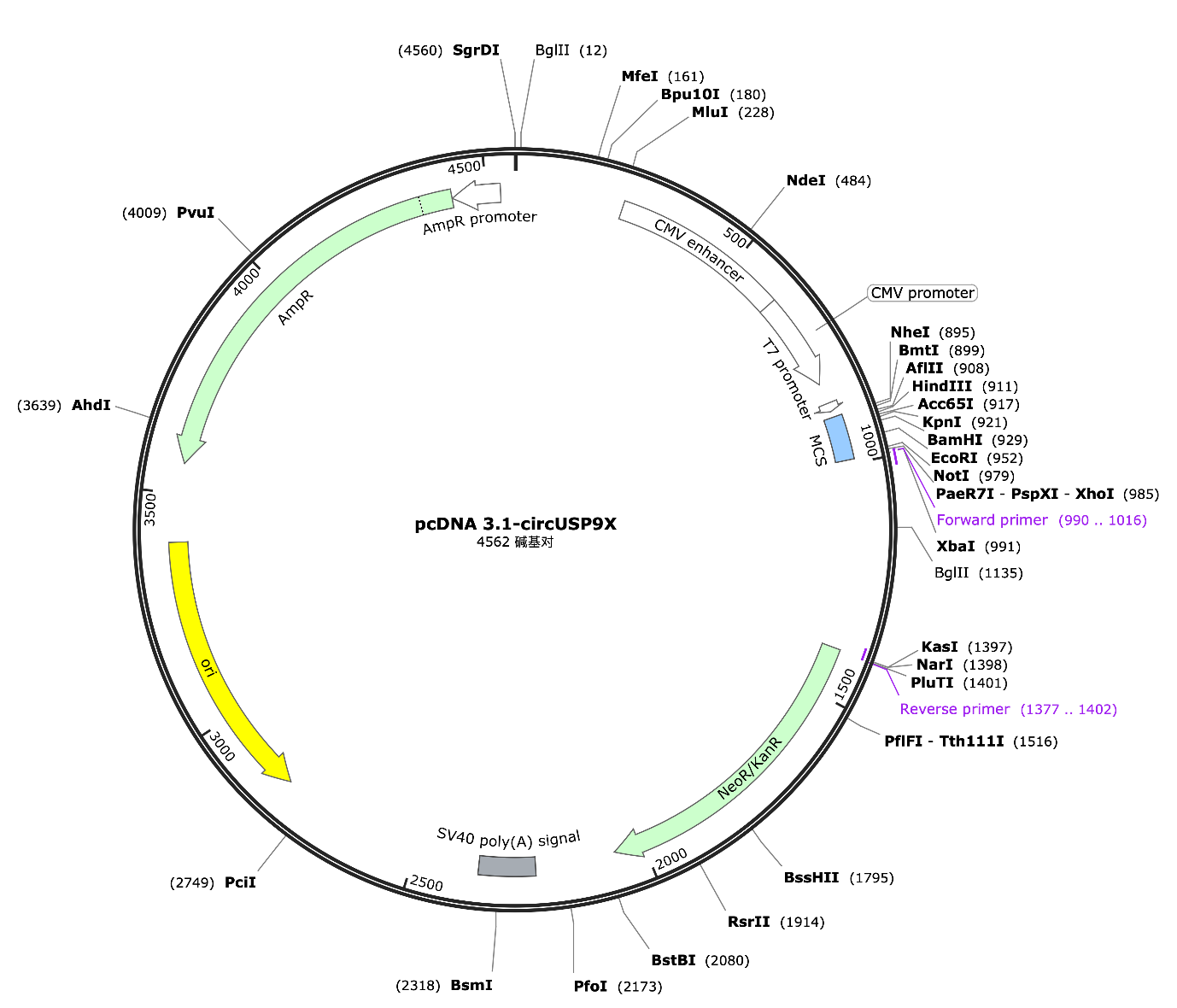

Supplement: Supplementary file 1 [file mmc1.docx]
